# Overexpression of SIRT6 attenuates the tumorigenicity of hepatocellular carcinoma cells

**DOI:** 10.18632/oncotarget.19297

**Published:** 2017-07-17

**Authors:** Yadong Wang, Teng Pan, Haiyu Wang, Li Li, Jiangmin Li, Ding Zhang, Haiyan Yang

**Affiliations:** ^1^ Department of Toxicology, Henan Center for Disease Control and Prevention, Zhengzhou 450016, China; ^2^ Henan Collaborative Innovation Center of Molecular Diagnosis and Laboratory Medicine, Xinxiang Medical University, Xinxiang 453003, China; ^3^ Department of Epidemiology, School of Public Health, Zhengzhou University, Zhengzhou 450001, China

**Keywords:** SIRT6, HCC cell, tumorigenicity, cell cycle

## Abstract

**Objective:**

This study aimed to explore the effects of overexpression of sirtuin 6 (SIRT6) on the tumorigenicity of hepatocellular carcinoma (HCC) cells

**Methods:**

Stable SIRT6-overexpressed HCC cell lines were established by transfecting SIRT6 plasmid. Soft agar assay and tumor xenograft assay in nude mice were applied. Flow cytometry was employed to detect cell cycle distribution. Western blotting analysis was used to detect the expression of proteins.

**Results:**

Overexpression of SIRT6 attenuated HepG2 and HCCLM3 cells proliferation, colony formation *in vitro* and tumor formation in nude mice, and resulted in the G1 phase cell cycle arrest. Overexpression of SIRT6 reduced the expression of cyclin D1 and p-ERK proteins in both HepG2 and HCCLM3 cells.

**Conclusion:**

Overexpression of SIRT6 attenuates the tumorigenicity of HCC cells.

## INTRODUCTION

Sirtuin 6 (SIRT6) is a member of the mammalian sirtuins family, which functions as a mono-ADP-ribosyltransferase and NAD^+^-dependent deacylase of both acetyl groups and long-chain fatty acyl groups [1]. SIRT6 is involved in multiple cellular processes including transcription [2], genome instability [3], telomere integrity [4, 5], DNA repair [6, 7], inflammation [8], metabolism and ageing [3, 9, 10], and cancer [11–14]. In the year of 2012, SIRT6 was identified as a novel tumor suppressor by regulating aerobic glycolysis in cancer cells [11].

Recently, a number of studies have identified SIRT6 as a key regulator of hepatocellular carcinoma (HCC), but the functional roles of SIRT6 in liver cancer are inconsistent. Min et al. reported that liver cancer initiation was controlled by AP-1 through SIRT6-dependent inhibition of survivin [15]. Marquardt et al. reported that loss of SIRT6 induced epigenetic changes which might be relevant to HCC development. Downregulation of SIRT6 and genes dysregulated by loss of SIRT6 possessed oncogenic effects in hepatocarcinogenesis [12]. Zhang et al. reported that overexpression of SIRT6 suppressed HCC cell proliferation and induced apoptosis [16]. Additional two studies [17, 18] suggested that SIRT6 acted as an oncogene in HCC development. Their results showed that SIRT6 was upregulated in a subset of HCC tissues and SIRT6 knockdown by shRNA suppressed the growth of HCC cells, induced apoptosis, and inhibited tumor growth of HCC cells *in vivo*. Therefore, the conflicts on the roles of SIRT6 in HCC promoted us to explore the functional roles of SIRT6 in the development of HCC and its underlying mechanisms. In this study, two stable SIRT6-overexpressed HCC cell lines were established to investigate the effects of overexpression of SIRT6 on HCC cell proliferation, cell cycle distribution, tumorigenicity *in vitro* and *in vivo*, and its related molecular mechanisms.

## RESULTS

### Identification of SIRT6 overexpression

**Figure 1:**
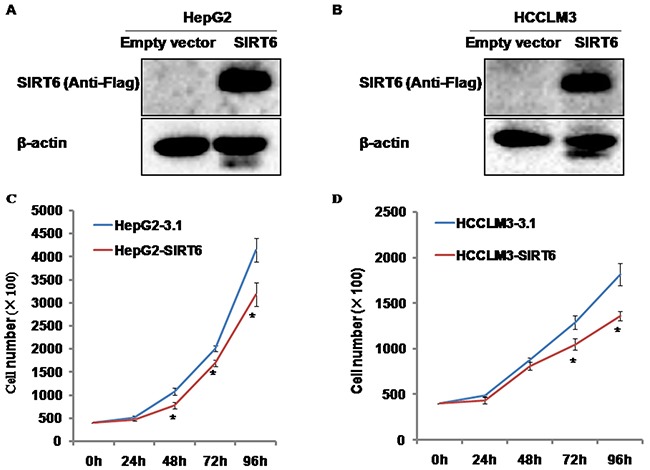
The identification of SIRT6-overexpressed HCC cells and the effects of SIRT6 overexpression on HCC cell proliferation (**A** and **B**) SIRT6 expression was only detected in HepG2-SIRT6 cells and HCCLM3-SIRT6 cells by anti-Flag, which suggests that stable SIRT6-overexpressed cell lines were successfully established. (**C** and **D**) Overexpression of SIRT6 decreased the proliferation of HepG2 cells from 48 hours and HCCLM3 cells from 72 hours. *P<0.05.

The overexpression of SIRT6 was identified by western blotting analysis. The expression of SIRT6 was only detected in HepG2-SIRT6 and HCCLM3-SIRT6 cells by using primary anti-Flag antibody, but not in HepG2-3.1 and HCCLM3-3.1 cells (Figure 1A and 1B), which suggests that stable SIRT6-overexpressed HCC cell lines were successfully established.

### Effects of SIRT6 overexpression on HCC cell proliferation

To test the effects of SIRT6 overexpression on HCC cell proliferation, we generated stable SIRT6-overexpressed HepG2 and HCCLM3 cell lines. The number of cells was counted at different time points indicated. SIRT6 overexpression significantly slowed down the growth of HepG2 cells from 48 hours and HCCLM3 cells from 72 hours compared with its controls, respectively (Figure 1C and 1D).

### Effects of SIRT6 overexpression on colony formation

**Figure 2:**
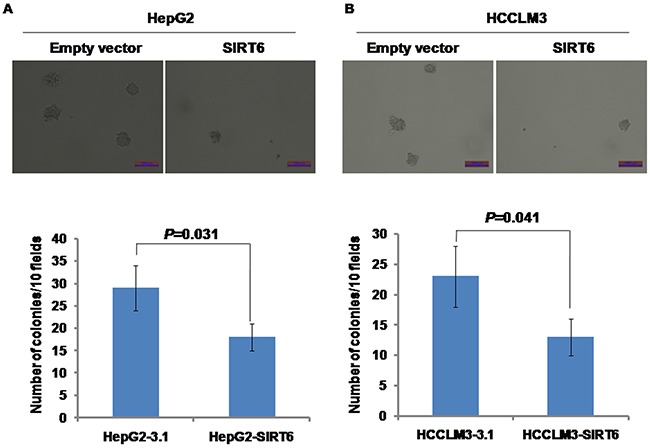
Effects of SIRT6 overexpression on colony formation Overexpression of SIRT6 significantly reduced the rate of colony formation in both HepG2 and HCCLM3 cells. The scale of bar is 1000μm.

Soft agar assay was employed to test the capability of colony formation of SIRT6-overexpressed HCC cells *in vitro*. We observed that overexpression of SIRT6 dramatically decreased the formation of cell colonies compared with its controls in both HepG2 and HCCLM3 cell lines (Figure 2A and 2B).

### Effects of SIRT6 overexpression on tumor growth in nude mice

**Figure 3:**
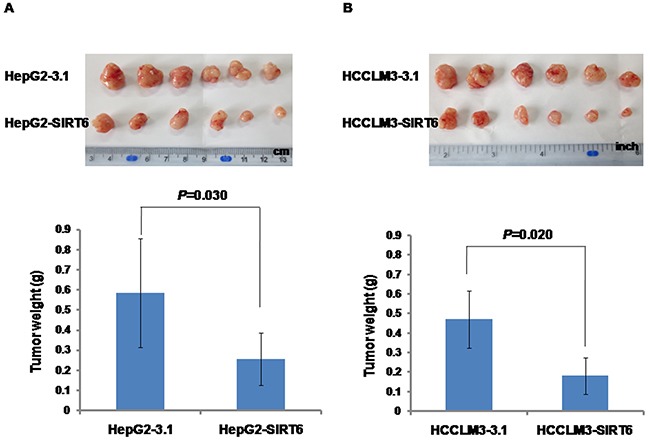
Effects of SIRT6 overexpression on tumor growth in nude mice Significantly decreased tumor weight was observed in the group injected with SIRT6-overexpressed HCC cells compared with the group injected with control cells.

To test the effects of SIRT6 overexpression on the capability of tumor growth *in vivo*, BALB/c nude mice xenograft models were established by subcutaneous injection with HepG2-SIRT6, HepG2-3.1, HCCLM3-SIRT6 and HCCLM3-3.1 cells. The tumor weight was significantly decreased in both HepG2-SIRT6 (0.2555 ± 0.1295 g) and HCCLM3-SIRT6 (0.1814 ± 0.0934 g) groups compared with HepG2-3.1 (0.5849 ± 0.2699 g) and HCCLM3-3.1 (0.4898 ± 0.1455 g) groups, respectively (Figure 3A and 3B), which suggests that overexpression of SIRT6 significantly reduced tumor formation and tumor growth of HepG2 and HCCLM3 cells *in vivo*.

### Effects of SIRT6 overexpression on cell cycle

**Figure 4:**
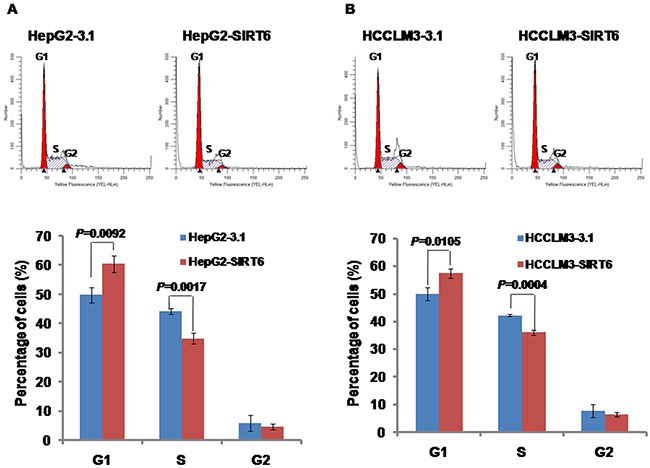
Effects of SIRT6 overexpression on cell cycle distribution The detection of cell cycle was performed in a Muse Cell Analyzer. Our data showed that overexpression of SIRT6 increased the cell percentage in the G1 phase and decreased the cell percentage in the S phase in both HCC cell lines.

Above, we observed that overexpression of SIRT6 decreased HCC cell proliferation. To further investigate whether or not the reduction of growth speed of SIRT6-overexpressed HCC cells is resulted from cell cycle arrest, the distribution of cell cycle was detected by flow cytometry. We observed the significantly increased proportion of cells stably overexpressing SIRT6 in the G1 phase population (60.5 ± 2.9% for HepG2-SIRT6 and 57.5 ± 1.7% for HCCLM3-SIRT6) compared with the control cells (49.9 ± 2.6% for HepG2-3.1 and 50.0 ± 2.3% for HCCLM3-3.1), respectively (Figure 4A and 4B). We observed the significantly decreased proportion of cells stably overexpressing SIRT6 in the S phase population (34.9 ± 1.9% for HepG2-SIRT6 and 36.1 ± 0.9% for HCCLM3-SIRT6) compared with the control cells (44.2 ± 1.0% for HepG2-3.1 and 42.2 ± 0.4% for HCCLM3-3.1), respectively (Figure 4A and 4B). Our results suggested that overexpression of SIRT6 might decrease HCC cell proliferation by arresting the cell cycle in the G1 phase.

### Overexpression of SIRT6 repressed the expression of cyclin D1

**Figure 5:**
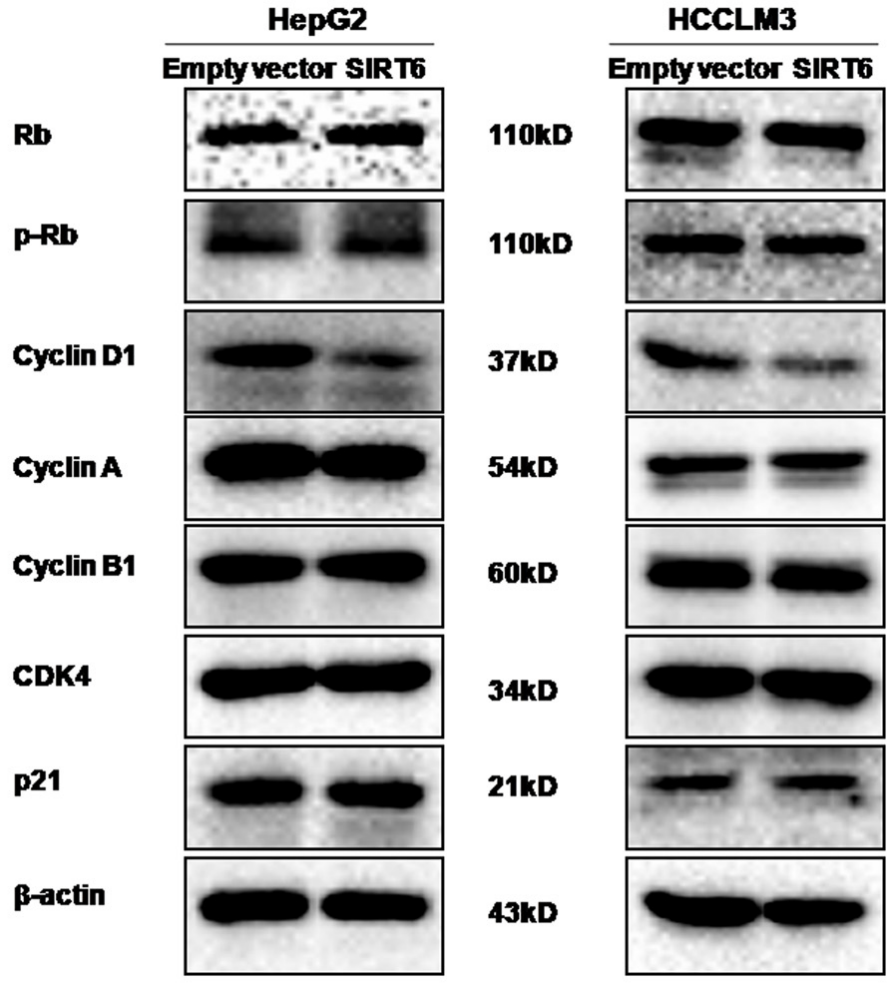
Effects of SIRT6 overexpression on the expression of cell cycle-related proteins The western blotting assay was performed. Our data showed that overexpression of SIRT6 could reduce the expression of cyclin D1 protein in both HCC cell lines.

To explore the molecular mechanisms of the cell cycle arrest while HCC cells stably overexpressing SIRT6, AQ:the expression of a series of cell cycle-related proteins was detected by western blotting analysis. Our data showed that the level of cyclin D1 protein expression was significantly decreased in HepG2-SIRT6 cells and HCCLM3-SIRT6 cells compared with its controls, respectively (Figure 5). There was no significant difference in the expression of the Rb, p-Rb, p21, cyclin A, cyclin B1 and CDK4 proteins between SIRT6-overexpressed HCC cells and its control cells (Figure 5).

### Overexpression of SIRT6 repressed p-ERK expression

**Figure 6:**
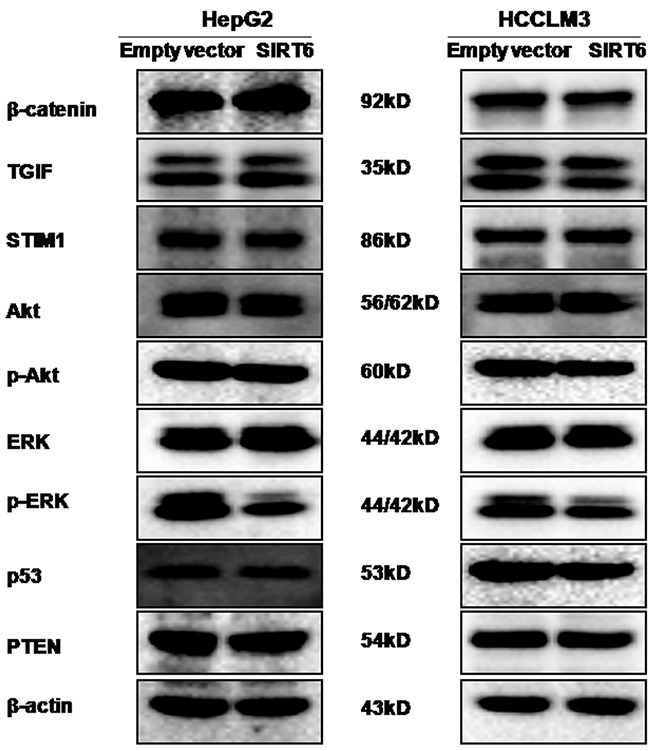
Effects of SIRT6 overexpression on the expression of several proteins The western blotting assay was performed. Our data showed that overexpression of SIRT6 could reduce the level of p-ERK protein expression in both HCC cell lines.

Since several proteins including β-catenin, TGIF, STIM1, p-Akt, p-ERK, p53 and PTEN are involved in HCC tumorigenesis, these proteins were detected by western blotting analysis. Our results showed that SIRT6 overexpression markedly reduced the level of p-ERK protein expression in both HepG2 and HCCLM3 cells (Figure 6). We did not observe obvious alterations in the expression of β-catenin, TGIF, STIM1, p-Akt, p53 and PTEN proteins between SIRT6-overexpressed HCC cells and its control cells (Figure 6).

## DISCUSSION

In this study, we observed that overexpression of SIRT6 suppressed the growth of HepG2 and HCCLM3 cells *in vitro*. Our observation is consistent with Zhang et al.'s results. Their results indicated that adenovirus-mediated overexpression of SIRT6 significantly inhibited HepG2 cell growth, whereas, silencing SIRT6 by shRNA promoted HepG2 cell growth [16]. But Marquardt et al. reported that overexpression of SIRT6 did not lead to a change in cell proliferation [12]. On the contrary, Lee et al. reported that SIRT6 knockdown by shRNA suppressed the growth of HCC cells (Huh7, SNU475 and SNU449) [17]. Ran et al. reported that depletion of SIRT6 inhibited the growth of liver cancer cell lines (PLC/PRF/5, SMMC-7721, Huh-7 and SK-Hep-1) [18]. The discrepancy among these studies should be addressed further among different laboratories in the future studies.

To further explore the underlying mechanisms of overexpression of SIRT6 in decreasing HCC cell proliferation, cell cycle distribution was analyzed by flow cytometry. Our data showed that overexpression of SIRT6 arrested cell cycle in the G1 phase in HepG2 and HCCLM3 cells. Moreover, we observed that overexpression of SIRT6 significantly decreased the expression of cyclin D1 protein in HCC cells, accompanied with the G1 phase arrest. Studies showed that the increased expression of cyclin D1 in cancer cells resulted in an uncontrolled growth advantage [19]. Cyclin D1 is required for progression through the G1 phase of the cell cycle, which dimerizes with CDK4/6 to regulate the G1/S phase transition and entry in the S phase. Cai et al. reported that DLK1 knockdown delayed the cell cycle G1/S transition in HCC cells, along with the decreased expression of cyclin D1 [20]. Duan et al. reported that suppression of ARID2 expression accelerated G1/S transition associated with upregulation of cyclin D1 in HCC cells [21]. Lee et al. reported that VRK1 knockdown increased the number of G1 phase arrested cells by decreasing cyclin D1 expression in HCC cells [22]. Taken together, our observations suggested that overexpression of SIRT6 arrested cell cycle in the G1 phase via decreasing the expression of cyclin D1 in HCC cells. But the mechanism underlying the regulation of cyclin D1 by SIRT6 was not addressed in this paper, which should be explored in the future study.

Previous study reported that SIRT6 promoted cell death during liver cancer initiation in diethylnitrosamine (DEN)-induced mouse liver cancer model [15], which suggests that SIRT6 might be a tumor suppressor in HCC tumorigenesis. To address the potential roles of SIRT6 in tumorigenicity of HCC cells, we established stable SIRT6-overexpressed HCC cell lines. Our data showed that overexpression of SIRT6 reduced the colony formation of HCC cells in soft agar and tumor growth in nude mice, which supported the opinion of SIRT6 as a tumor suppressor in HCC to a certain extent. However, other two studies reported opposite results. Lee et al. reported that SIRT6 depletion reduced colony formation of HCC cells in soft agar and tumor growth in xenograft mouse model [17]. Feng et al. reported that upregulation of SIRT6 was required for TGF-β1/H_2_O_2_/HOCl to promote the tumorigenicity of HCC cells owing to that suppression of SIRT6 expression could abrogate the promoting effects of TGF-β1/H_2_O_2_/HOCl on the tumorigenicity of HCC cells [23].

The extracellular signal-regulated kinase (ERK) pathway is involved in crucial cellular processes, including proliferation, differentiation, angiogenesis and survival [24]. Overexpression or activation of ERK is believed to contribute to hepatocarcinogenesis. Studies have shown that an increase in the expression and activity of ERK was found in human HCC tissues [25–27] and in a rat model of experimental HCC [28]. ERK knockdown abolished liver tumor cell proliferation and DNA replication as well as the growth of xenografted tumors [29]. In this study, overexpression of SIRT6 decreased the level of p-ERK expression in two HCC cell lines. Consistent with our observations, one published paper reported that overexpression of SIRT6 significantly inhibited ERK1/2 phosphorylation in HCC cells [16]. Both of these studies suggested that the ERK signaling pathway might be involved in SIRT6 suppressing the proliferation and tumorigenicity of HCC cells.

In conclusion, our data suggested that overexpression of SIRT6 reduced HCC cell proliferation by arresting cell cycle in the G1 phase and attenuated the tumorigenicity. Therefore, this study extends our knowledge of liver tumorigenesis.

## MATERIALS AND METHODS

### Cell culture

The cell lines of HepG2 and HCCLM3 were purchased from the Cell Resource Center, Peking Union Medical College (which is the headquarters of National Infrastructure of Cell Line Resource, NSTI). The two cell lines were cultured in DMEM medium containing 10% of fetal bovine serum (FBS), 2 mM of L-glutamine, 100 U/ml of penicillin, and 100 μg/ml of streptomycin at a condition of 37°C and 5% CO_2_.

### Establishment of stable cell lines

The enhanced SIRT6 plasmid with flag tag was obtained from Addgene (#13817) and the empty vector (pcDNA3.1) was purchased from Invitrogen (V79020). The transfection was performed using Lipofectamine 2000 according to the manufacturer's instructions (Invitrogen, USA). The transfected cells of HepG2 and HCCLM3 were selected with 500μg/ml of G418 (Gibco, USA). The stable cell lines were identified by western blotting and named as HepG2-SIRT6 and HepG2-3.1, and HCCLM3-SIRT6 and HCCLM3-3.1, respectively.

### Detection of cell proliferation

The assay of cell proliferation was performed as previously described [30]. In brief, 4 × 10^4^ cells were plated into each well of 12-well plates in triplicate. Cells were trypsinized and counted by using a CASY Cell Counter (Scharfe System, Reutlingen, Germany) at 24, 48, 72, and 96 hours after plating.

### Soft agar assay

The assay of soft agar was performed as previously described [31]. 500 cells in 1 ml of DMEM full medium containing 0.35% low-melting point agarose were plated on top of the base layer (3 wells per group). Cells were maintained at a condition of 37°C and 5% CO_2_ for two weeks.

### Tumor xenograft assay

This study was approved by the Ethics Committee of Henan Center for Disease Control and Prevention. All of the animal experiments were carried out according to the Guide for the Care and Use of Laboratory Animals. Male BALB/c nude mice aged four weeks were obtained from Vital River Laboratory Animal Technology Co., Ltd. (Beijing, China). Cells were trypsinized and washed with PBS. 5 × 10^6^ cells in 150 μl of PBS were subcutaneously injected into the back neck of each mouse (six mice per group). The mice were monitored every day for tumor formation and were sacrificed at 18 days postinjection [31].

### Western blotting assay

The western blotting analysis was performed as previously described [30]. In brief, cells were lysed by RIPA buffer containing protease inhibitors and phosphatase inhibitors (Pierce, USA). The protein concentration was measured by a BCA regent kit (Pierce, USA). The lysates were loaded into 10% of sodium dodecyl sulfate-polyacrylamide electrophoresis gels and then transferred to nitrocellulose (NC) membranes (PALL, USA). The membrane was blocked with 5% of bovine serum albumin (BSA) in Tris-buffered saline-Tween 20 and then incubated with a primary antibody at 4°C for overnight. The anti-Flag (F3165) was purchased from Sigma. The β-catenin (sc-7199), TGIF (sc-9084), p21 (sc-397), STIM1 (sc-68897), p53 (sc-6243), Akt (sc-8312), p-Akt (sc-33437), cyclin A (sc-751), cyclin B1 (sc-752), cyclin D1 (sc-718), CDK4 (sc-260), and β-actin (sc-8432) were purchased from Santa Cruz Biotechnology. The Rb (#9313S), phospho-Rb (#8516S), ERK1/2 (#4695S), p-ERK1/2 (#4370S), and PTEN (#9188S) were purchased from Cell Signaling Technology. The secondary antibodies (Peroxidase-Conjugated AffiniPure Goat Anti-Rabbit-IgG and Goat Anti-Mouse-IgG) were purchased from ZSGB-BIO (Beijing, China). The signals were detected in the ChemiDoc™ XRS+ Imaging System (Bio-Rad, USA) by a Bio-Rad Clarity™ western ECL substrate (Bio-Rad, USA).

### Detection of cell cycle

Cells were plated in 60-mm dishes, and trypsinized while growing to 70-80% confluence. Detached cells were harvested, washed with PBS and fixed in 70% of ethanol at −20°C overnight. The detection of cell cycle was performed in a Muse Cell Analyzer (Millipore, Merck Group) by using Muse cell cycle assay kit (MCH100106, Millipore) according to the manufacturer's instructions.

### Statistical analysis

The Student's *t* test was performed to estimate the statistical significance by using the SPSS 13.0 software (SPSS, Chicago, IL). Values were presented as mean ± standard deviation (SD). Difference was considered statistically significant when *P* < 0.05. All of the tests were two-sided.
